# The Impact of Environmental Regulation on Firm Performance: Evidence from the Pulp and Paper Industry in China

**DOI:** 10.3390/ijerph20042982

**Published:** 2023-02-08

**Authors:** Xu Ou, Haiwei Jiang

**Affiliations:** 1 School of Economics, Zhejiang University, Hangzhou 310058, China; 2 School of International Trade and Economics, Central University of Finance and Economics, Beijing 100081, China

**Keywords:** environmental regulation, firm performance, special emission limit, reallocation effect, inventory alleviation effect

## Abstract

In areas with serious pollution problems, the government designates a special emission limit (SEL) for pollution control and environmental protection in China. This paper examines the effects of chemical oxygen demand (COD) SEL on firms’ production activity and market performance in the pulp and paper industry in the Lake Tai area in China. Using firm-level data, we employ a difference-in-differences strategy and find that SEL has a negative impact on the production scale, profitability, and market size of the regulated firms, while showing no significant impact on firm exports. The heterogeneity tests suggest that the impact of SEL on production and market performance varies with firm ownership, firm size, and target market. The reallocation effect of production shifts extra production from exited firms to existing firms, which explains the expansion of production scale and market size for SOEs and large-sized regulated firms. Compared with the decline of production scale, the inventory alleviation effect reduces the negative impact of stricter environmental regulation on firm performance.

## 1. Introduction

In the past few decades, fast-growing countries have been going through the stage of balancing economic development with severe environmental issues. In order to alleviate the pressures of environmental challenges, countries enact environmental regulations worldwide. These regulations affect economic development in many ways, such as technological innovation, resource utilization, and firm production. As the largest developing country, China started enacting stringent environmental regulations for different pollutants due to environmental deterioration [1,2,3]. Government intervention with environmental policy impacts affected firms’ behaviors and outcomes. A question is: To what extent and how does environmental regulation affect firm performance in China?

To answer this question, this paper studies the effect of environmental regulation on firm performance using the policy change of wastewater discharge. We first conduct an empirical analysis with the refined identification strategy. The implementation of special discharge limits (SEL) for water pollutants, was announced by the Ministry of Environmental Protection of China in 2008. SEL is more stringent than the general emission limit in one pollutant discharge standard, which requires regulated firms to emit fewer pollutants. Firms are asked to stop production by the local environmental department if they fail to meet the standard. Thus, it provides a natural experiment for empirical analysis of the impact of command-and-control environmental regulation on firm production activities and market performance. Our identification strategy is based on a difference-in-difference framework that compares changes in firm-level production scale and market performance over time for firms in the pulp and paper industry between the Lake Tai area and other areas in Jiangsu, Zhejiang, and Shanghai. Using the data from the Annual Survey of Industrial Firms (ASIF) during 2003–2013, the empirical results suggest that the special emission limit reduces production scale, profitability, and market size by 9.5%, 9.1%, and 8.0%, respectively, for firms in the pulp and paper industry in the Lake Tai area, compared with those in the control group. We conduct various robustness checks, such as examining common trend assumption, expanding the range of regulated industries in the sample, and applying different model specifications. The results are quite robust. We further examine the heterogeneous effects of SEL associated with firm ownership, size, and market base. We find that SEL significantly increases production scale, profitability, and market size for stated-owned and large-sized firms in the pulp and paper industry, while decreasing the production scale and market performance for non-stated-owned and small-sized firms. However, SEL had no statistically significant effect on overall exports for firms in the pulp and paper industry in regulated districts.

We provide two potential explanations for the heterogeneity effect of SLE on production scale and market performance for different types of firm ownership, size, and target market. The first one is the reallocation effect of production. After implementing SEL in the Lake Tai area, non-stated-owned and small-sized firms reduce production scale, and the production unfinished by regulated firms is released to the market. Since stated-owned and large-sized regulated firms are more capable of absorbing the released production, our results suggest that stated-owned and large-sized regulated firms expanded their production scale and market size after implementing SEL. The second one is the inventory alleviation effect. Market performance is affected by not only regulation but also inventory. Though stricter environmental regulation reduces firm production scale, the regulated firm can obtain profits by selling inventory. Therefore, having inventory offsets the negative impact of SEL on market performance.

This paper contributes to the literature in two ways. First, this paper contributes to the limited empirical evidence on the impact of wastewater pollutant discharge standards in emerging economies. A growing body of literature has discussed the impact of environmental regulation on the environmental and economic outcomes in China, such as pollution reduction mandates [4,5,6], river chief system [7,8,9], and central supervision [10,11,12,13]. However, only a few researchers pay attention to the impact of discharge standard changes, which directly affect pollution emissions and the economic performance of manufacturing firms. Two exceptions are Liu et al. (2017) and Zhang et al. (2020) [14,15]. The former studies the impact of a wastewater discharge standard on the labor demand of the textile, printing, and dyeing (TPD) industry, and their result shows that the discharge standard reduced labor demand by 7% [14]. The latter also focus on the TPD industry and find that the wastewater discharge standard through the mechanism of entry-exit in the export market, adjustments of exporting destinations and product reduce the export probability and scale of TPD firms in Jiangsu province compared with other provinces within the Lake Tai area [15]. We extend these empirical studies by providing evidence of the economic impact of the pollutant discharge standard on firm performance.

Second, this paper provides one of the first pieces of evidence on the reallocation effect of production across firms within the same regions. Previous literature has investigated the impact of water environmental protection regulations on pollution emission [13,16,17,18] and firm productivity [10,19], while a few researchers focus on the reallocation effect of firm production. Most of the existing literature focuses on the reallocation effect of pollution [20,21], capital [4,22], and labor demand [14,23,24] across regions, but little pay attention to the reallocation effect of production across firms within the same region [25]. Prior studies find that the reallocation effect of production from regulated to unregulated firms appears not only within the same regulated industries [25,26], but also within the same conglomerate [27]. This paper is devoted to previous studies documenting evidence of the reallocation effect across regulated firms within the same regulated industries and areas.

The remainder of the paper is organized as follows. Section 2 introduces the institutional background of COD emission limits for the pulp and paper industry in the Lake Tai area of China. Section 3 describes the data. Section 4 presents the empirical methodology and results. Section 5 concludes.

## 2. Institutional Background: COD Emission Limits for the Pulp and Paper Industry in the Lake Tai Area of China

The rapid growth of the economy and the degradation of water quality resulted from rapid industrialization. From the mid-1990s to the early 2000s, pollutants in industrial wastewater caused the water quality of the Lake Tai to degrade from class II to class IV water standard [15,28]. Industrial wastewater contains a large number of organic substances, such as nitrogen and phosphorus, which lead to algal blooms [29]. The algal blooms consume a large amount of dissolved oxygen in the water, making other organisms, such as fish and decomposers die due to the difficulty in obtaining oxygen. Due to the lack of sufficient decomposers, it is difficult for the carcasses in water to be decomposed in time. The undecomposed carcasses in water not only produce odor, but also produce harmful substances such as humus. The harmful substances increase the probability of cancer in the normal cells of organisms (including human beings) [30,31,32]. In addition, the genes of algae organisms may mutate into toxic algae under the action of harmful substances, secreting toxic substances, making the sewage into toxic water and endangering the ecosystem [33].

To deal with the serious water pollution problem in the Lake Tai, the central and local governments have tightened regulations on the discharge of water pollutants from industrial firms. On January 1 of 2008, the Department of Environmental Protection of Jiangsu province implemented a local water pollutant discharge standard for urban sewage treatment plants and six key industries (DB32/1072-2007), including two types of pulp and paper firms, in the Lake Tai area located in Jiangsu province. On 25 June 2008, the Ministry of Environmental Protection (MEP) of China enacted a new national discharge standard of water pollutants for the pulp and paper industry (GB3544-2008), which was implemented on 1 August 2008. In July 2008, MEP announced that the industries and administrative areas would implement the special emission limit for water pollutants discharge. On 1 September 2008, firms in the pulp and paper industry in the Lake Tai area implemented SEL of GB3544-2008. GB3544-2008 is implemented for new pulp and paper firms established after 1 August 2008 from the date of establishment, while it sets a buffer time for old pulp and paper firms established before 1 August 2008. In theory, old pulp and paper firms implement the policy in three phases. In the first phase before 1 May 2009, they implemented the old standard GB3544-2001. In the second phase starting on 1 May 2009, old firms implemented buffer emission limits stated in GB3544-2008. The buffer emission limits are more stringent than GB3544-2001. In the third phase starting on 1 July 2011, old firms implemented GB3544-2008. In particular, all pulp and paper firms, no matter what the date of establishment is, in the Lake Tai area implemented GB3544-2008 after 1 September 2008.

The Lake Tai area in our analysis refers to the administrative areas stipulated in the 2008 document No. 30 of the Ministry of Environmental Protection, including Jiangsu, Zhejiang, and Shanghai, as shown in Table 1. Only Jiangsu province has implemented a local standard to discharge water pollutants in the Lake Tai area for the pulp and paper industry, and the regulated regions in the 2007 Jiangsu local standard (DB32/1072-2007) belong to the regions in Table 1. There could be two levels of wastewater discharge standards, namely, the national standard and the local standard. In fact, firms are obligated to comply with the stricter one in China. In other words, if the local standard is stricter than the national one, the firm implements the local one. If the national standard is stricter than the local one, the firm still implements the national standard.

DB32/1072-2007 only implemented stricter COD emission limits than the national standard GB3544-2001 for pulp and paper-making firms using wastepaper pulping technologies and the paper-making firms using commercial pulp. Comparing the emission limits implemented in the pulp and paper industry in DB32/1072-2007 and the special emission limits in GB3544-2008, it is found that the limits for ammonia nitrogen (NH_4_^+^-N) and total phosphorus (TP) are the same, while the limits implemented in GB3544-2008 for chemical oxygen demand (COD) and total nitrogen (TN) are more stringent than the ones in DB32/1072-2007. COD is the most widely used comprehensive indicator to measure the concentration of organic substances in the environmental regulation of water [34]. The greater the value of COD, the more serious the water pollution. COD has the most significant impact on firm performance compared with other regulated indicators [11]. Stricter environmental regulations on COD emission intensity have raised the compliance cost of regulated firms by investing in environmentally-friendly inputs and products [35], as well as cost-saving technologies and advanced end-of-pipe treatment technologies in the short term is consistent with the Porter hypothesis.

Appendix A Table A1 shows the COD emission limits of regulated firms in the pulp and paper industry in the Lake Tai area of Jiangsu Province at different times, and lists the general COD emission limits of firms in the pulp and paper industry in other unregulated areas. From1 January 2008 to 1 August 2008, in the areas regulated by DB32/1072-2007, the COD emission limits decreased from 150 mg/L to 100 mg/L and from 100 mg/L to 80 mg/L for pulp and paper-making firms using waste paper deinking technologies and the paper-making firms using commercial pulp, respectively, while the COD emission limits implemented by other types of firms in the pulp and paper industry remain unchanged. Compared with the general COD emission limits for old and regulated firms in the pulp and paper industry in GB3544-2008 in the first and second phases, DB32/1072-2007 is not a stricter standard. The COD emission limits for the pulping firms are decreased to 100 mg/L, and the range of decline is 71.43–77.78% for the pulping firms with different production processes. The COD emission limits for the pulping and paper-making firms are decreased to 90 mg/L, and the decline range is 74.29–80.00% for the pulping and paper-making firms with different production processes. The COD emission limits for the paper-making firms are decreased to 80 mg/L, in which the COD emission limit is decreased from 100 mg/L to 80 mg/L for the paper-making firms using wastepaper and remains unchanged at 80 mg/L for the paper-making firms using commercial pulp. Therefore, firms in the pulp and paper industry in the areas regulated by DB32/1072-2007 have implemented GB3544-2008, which is more stringent than the local standard, since 1 August 2008. Conversely, DB32/1072-2007 is not a stricter standard for the new and regulated firms after 1 August 2008, and the old and regulated firms in the third phase. Among GB3544-2001, DB32-1072-2007, and GB3544-2008, GB3544-2001 is the least stringent standard and GB3544-2008 is the most stringent standard.

The special emission limits aim to strictly control firms’ discharge behaviors to protect the ecological environment in areas prone to severe water pollution problems. Thus, the special emission limit is more stringent than the general emission limit in one pollutant emission standard. Compared with the general COD emission limit in GB3544-2008, SEL is 80 mg/L for the pulping firms, which is 20.00% stricter than the general emission limit. SEL is 60 mg/L for the pulping and paper-making firms, which is 33.33% stricter than the general emission limit. Meanwhile, SEL is 50 mg/L for the paper-making firms, which is 37.50% stricter than the general emission limit. Therefore, SEL is the most stringent water pollutants discharge standard.

We focus on the impact of SEL on firms in the pulp and paper industry in the Lake Tai area. The interference of DB32/1072-2007 and GB3544-2008 is negligible. First, it is difficult to observe the effect of the policy implemented for only a few months in annual data. DB32/1072-2007 was replaced by GB3544-2008 eight months after implementation and the general COD emission limits in GB3544-2008 were replaced by SEL only one month after implementation, so the interference of these two standards is limited. Second, the number of sub-industries affected by the inference standards is limited. DB32/1072-2007 only interferes with the impact of SEL on the pulping and paper-making firms using wastepaper deinking technologies. Due to restricted information, it cannot identify the number of pulping and paper-making firms using wastepaper deinking technologies.

Based on the above discussions, our empirical analysis focuses on firms in the pulp and paper industry in the Lake Tai area. The firm-level observations enable us to investigate the impact of SEL on firm performance.

## 3. Data and Methodology

### 3.1. Data

The pulp and paper industry firms used in our analysis are identified according to the documents issued by the Ministry of Environmental Protection in 2008 (Ministry of Environmental Protection No. 28 and No. 30 of 2008). The data relating to firm characteristics, production activities, and financial statements are obtained from the Annual Survey of Industrial Firms. ASIF documents comprehensive information on state-owned and non-state-owned industrial firms, which is conducted by China’s National Bureau of Statistics (NBS). ASIF is the most extensive firm-level dataset utilized in research on China’s microeconomics [10,14,25,36,37,38].

In order to construct consecutive panel data, we merge the datasets of ASIF across years using firm-level ID, name, representative name, main products, telephone, postal code, and year of establishment [36]. As SEL in the Lake Tai area was implemented in the middle of 2008, to avoid the disturbance of China’s entering into WTO at the end of 2001 and the SEL in the Pearl River Delta after 2013, the sample period is chosen from 2003 to 2013. To deal with outliers, samples that violated accounting standards and lacked core indicators are excluded [37]. All monetary values, including total output, sales, income, and exports are deflated to 2001 using the ex-factory price indices. The final number of observations of the merged data is 18,080 (4953 and 13,127 observations in our treatment and control groups, respectively) from 2003 to 2013, spanning five years before the implementation of SEL (2003–2007) and six years after the implementation of SEL (2008–2013).

Table 2 shows the descriptive statistics for the variables used in the analysis. Before the implementation of SEL, the mean of output, sales, income, and exports in the treatment group is larger than that in the control group, while after the 2008 SEL, the mean of all four dependent variables in treatment is close to that in the control group. The difference in all four indicators between the treatment and control groups is smaller after the 2008 SEL. The software for statistical analysis we use in this paper is Stata 16.0.

### 3.2. Difference-in-Difference Strategy

As mentioned, the special discharge limits set their wastewater discharge requirements for regulated regions. Thus, firms of regulated industries in Jiangsu, Zhejiang, and Shanghai can be classified into two groups due to their location. Firms of regulated industries in the Lake Tai area faced more stringent environmental regulation (i.e., treatment group) than those in non-Lake Tai areas (i.e., control group). In addition, county-level environmental regulation is the lowest level of China’s administrative governance of the environment. Hence, firms of the same regulated industry in the same county face the same intensity of environmental enforcement.

Utilizing the natural experiment brought by wastewater discharge change, we adopt a difference-in-difference approach to investigate the effect of the special emission limits on the performance of firms in the pulp and paper industry in the Lake Tai area. The empirical specification is as follows:

Y_ict_ = β_0_ + β_1_Treat_c_ × Post_t_ + γ’**X**_it_ + D_i_ + D_t_ + D_c_ + ε_ict_ (1)

where Y_ict_ is the measure of firm i’s performance in county c in year t. We measure firm performance using four variables, total output, sales, income, and exports. Accordingly, these four variables capture a firm’s production scale, profitability, overall market scale, and overseas market scale. To mitigate the effect of heteroscedasticity on the estimation results, all absolute numeric values are treated in logarithmic form. Treat_c_ is equal to 1 if firm i located in the Lake Tai area; otherwise, it is equal to 0. Post_t_ is equal to 1 for years after 2008 (the treatment period); otherwise, it is equal to 0. Treat_c_ × Post_t_ is the interaction term that captures the average differential change in firm performance in the treatment group relative to the control group during the treatment period (2008–2013). The interest of our research is β_1_, if it is statistically significant and negative, we can conclude that SEL forces firms’ scale to collapse. **X**_it_ refers to a group of firm characteristic variables that might impact firm performance, including firm size, ownership, and age. Firm size is one of the typical characteristics of a firm. Large-sized firms have more capital and larger market shares, and thus are easier to have better economic performances than small-sized firms [39]. Firm size is measured by the number of employees. Due to the stronger connection with local government, SOEs tend to achieve better economic performances than non-SOEs in resource-intensive industries [40]. Ownership is characterized by the type of registration. Firm age affects profitability [41,42,43], and it is calculated as the statistical year subtracting the year of establishment. D_i_ is the firm fixed effects, D_t_ is the year fixed effects, D_c_ is the county fixed effects. The firm and county fixed effects are included to control for the firm- and county-specific features that are time-invariant, and the year fixed effects are used to control for macroeconomic factors that affect all firms over time. ε_ict_ is the stochastic error term.

## 4. Empirical Results

### 4.1. Baseline Results

Our baseline results for the DID analysis are based on Equation (1). Table 3 presents the main results of the regressions with different firm-level dependent variables, including total output, sales, income, and exports. The coefficients of the interaction term of SEL are statistically significant and negative at the 1% level for total output, sales, and income, whereas the coefficient of the interaction terms is not statistically significant for exports. The results imply that the production scale, profitability, and market scale of firms in the pulp and paper industry in the Lake Tai area facing stricter water pollutants discharge standards decreased significantly after the implementation of SEL in 2008, by 9.5%, 9.1%, and 8.0%, respectively, compared with the control group, while the export scale is not affected by SEL. The R-squared values in all four models are above 0.83 in our analysis, meaning that the empirical model fits the data well. In addition, the number of observations is above 15,000, which indicates that the results have general implications. Overall, these results indicate that the SEL has a significant and negative effect on firm performance.

### 4.2. Test for the Common Trend Assumption

Satisfying the common trend assumption is the precondition of the results of the DID model that accurately captures the causal effect of SEL on firm performance. The common trend assumption states that the treatment group follows the same trend as the control group before implementing the policy. We test this assumption using the event study as follows:

(2) $${\;Y}_{\mathrm{ict}}=\alpha_{0}+\sum_{t=2003}^{2013}\alpha_{t}\left[{\mathrm{Treat}}_{c}\times 1\left[{\mathrm{year}}_{t}\right]\right]+\delta X_{\mathrm{it}}+D_{i}+D_{t}+D_{c}+\varepsilon_{\mathrm{ict}}$$

where α_t_ captures the differential changes between firms in the treatment and control group before and after the implementation of SEL in 2003–2013. Following previous literature [44], we select 2007 as the baseline year (one year before the implementation of SEL).

**Figure 1:**
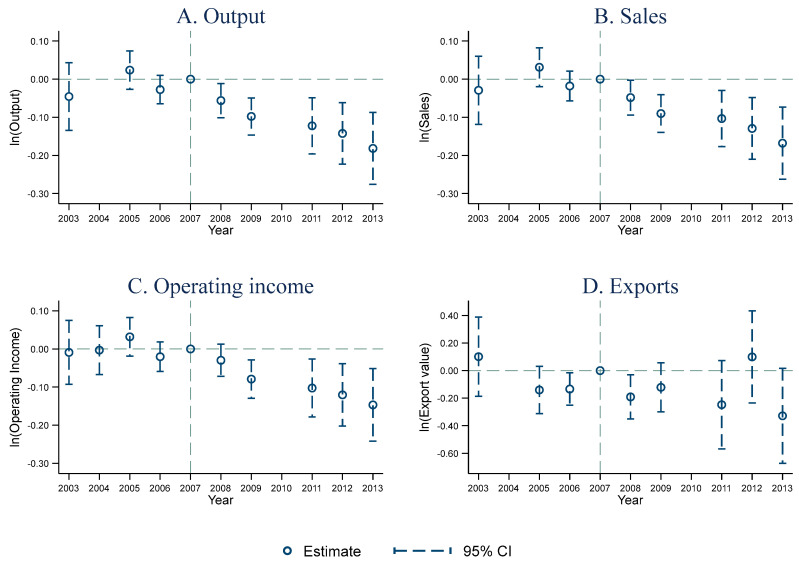
Common trend tests for the impact of SEL on firm performances.

Figure 1 illustrates that the differences in mean values of all four dependent variables between the treatment and control groups are not statistically significant before the implementation of SEL (2003–2006). The findings imply that the differences in all four dependent variables between the treatment and control groups satisfy the common trend assumption.

After the implementation of SEL, the differences in production scale, profitability, and overall market scale between the treatment and control groups significantly decrease, and the differences in exports between the treatment and control groups are still significantly unchanged. Moreover, the production scale of the firms in the Lake Tai area decreases significantly from 2008, which is the year of SEL’s implementation, and the differences in profitability and overall market scale are still unchanged until 2009. One potential explanation is that SEL immediately affects the production activities of the firms in the Lake Tai area, while it has a lagging effect on sales.

Overall, the test presented in Figure 1 is consistent with the baseline regression results, further ensuring the validity of the DID results.

### 4.3. Robustness Checks

The above analysis employs the pulp and paper industry as the treatment group because it is exactly regulated under the environmental policy as mentioned. To alleviate the plausible bias caused by other related industries, we use all 12 industries regulated by DB32/1072 or SEL in the national standards for water pollutants discharge standards as the treatment group to conduct robustness checks. The results are presented in Table 4. The coefficients of the DID interactions are negative and significant at the 1% level from column (1) to column (3), and the coefficient of DID interaction is insignificant in column (4). All results are consistent with the baseline results, suggesting that the results are robust.

To address the concern that other time-varying factors at the region and industry level might have impacts on our empirical results, we include other fixed effects as robustness checks. We consider province-year fixed effects to control for differential province-specific time effects such as macro shocks and 4-digit industry-year (CIC4-year) fixed effects to control for differential industry-specific time effects such as technology innovation within industries. The results are presented in Table 5. Columns (1) to (4) are the results that control province-year fixed effects, while columns (5) to (8) are the estimates that control industry-year fixed effects. It shows that the results are robust after controlling for different fixed effects.

### 4.4. Heterogeneous Tests

So far, we have concluded that stricter wastewater discharge standards negatively impact firm performance. We seek to provide two reasonable explanations for the findings. (i) Reallocation effect. Strict environmental regulation can improve resource allocation due to the exit of unproductive firms [26,45]. It means that production from exited firms to existing firms. In particular, market share due to the withdrawal of regulated firms in the Lake Tai area may be absorbed by the existing firms inside or outside the region. As a result, some existing firms that obtain extra demand may expand their production scale in the Lake Tai area. (ii) Inventory alleviation effect. Inventory contributes to direct and indirect costs, but the optimal strategy for a firm is to carry strategic inventories in any circumstance [46,47]. Although the regulated firms no longer produce new products during the stop-production period, they have income by selling inventory products. Therefore, SEL’s negative impact on the market performance is alleviated.

In this section, we test firm heterogeneities to examine the reallocation effect and inventory alleviation effect. The heterogeneous effects of SEL on production activities and market performance are explored in three dimensions, including firm ownership, size, and income source.

#### 4.4.1. Firm Ownership

Based on the type of firm registration, firms can be categorized into state-owned enterprises (SOEs) and non-state-owned enterprises (non-SOEs) in China. Compared with non-SOEs, SOEs are more likely to be subject to government intervention and expected to fulfill multiple targets beyond profit making, including employment, taxable income, structural change, and local growth targets that are engaged based on the local economic situation [48,49]. Therefore, the profit maximization functions between SOE and Non-SOE firms are different, and the impacts of SEL’s implementation on the regulated firms’ production activities and market performance could be heterogeneous.

Table 6 presents the DID estimation results for different ownership. When the dependent variables are the logarithm of output, sales, and income, the coefficients of the 2008 SEL are positive and significant for SOEs, while they are negative and significant for non-SOEs. The findings demonstrate that, after SEL’s implementation, the production and sales income in the Lake Tai area are transferred from non-SOEs to SOEs. SOEs have closer political ties with local governments than non-SOEs, and thus bear lower environmental risks [50]. Therefore, faced with the same intensity of environmental regulations, the negative impacts of SEL on the production activities and market performance for SOEs are less than that for non-SOEs.

Whether SOEs or non-SOEs, the decline of the regulated firms’ sales in the Lake Tai area is less than that of output, indicating that some factors diminish the negative impact of SEL on the sales. The result provides a clue that firm inventory is a key factor as mentioned. Furthermore, consistent with the benchmark regression results, SEL has no significant impact on the exports of both SOEs and non-SOEs.

#### 4.4.2. Firm Size

Facing environmental regulation, regulated firms invest in upgrading production and (or) sewage treatment technologies to meet SEL and avoid exiting the market. The mechanism of the impact of stringent environmental regulation on firm performance varies across firm sizes. Compared with small-sized firms, large-sized firms are more likely to use green production technologies and advanced end-of-pipe treatment technologies that require large installation costs [51,52]. When environmental regulations are tightened, small-sized firms tend to take longer to raise funds to upgrade technologies, so they are much more likely to be shut down than large and medium-sized firms. Furthermore, the production from exited firms would be absorbed by the existing firms. Compared with medium-sized firms, large-sized firms have more sophisticated production technologies to complete extra production that is returned to the market due to the exit of firms. We expect that the impact of SEL is negative for medium and small-sized regulated firms and probably positive for large-sized regulated firms. To answer the question, we analyze the heterogeneous effect of SEL on production scale and market performance based on firm size.

The size of Chinese industrial firms is classified into four types: large, medium, small, and micro-sized according to the number of employees, business income, and total assets (the National Bureau of Statistics, 2003, 2011), as shown in Appendix A Table A2. Considering the consistency of standards and data availability, we categorize all firms into three types of size, large, medium, and small-sized firms (including micro-sized firms), based on the 2011 NBS classification standard.

Table 7 shows the results of the heterogeneity analysis based on firm size. Compared with firms in the pulp and paper industry in the control group, the coefficients of SEL are positive and significant for large-sized regulated firms, but negative and significant for small-sized regulated firms, and are insignificant for medium-sized regulated firms. These results reassure us that the production scale and sales partly transfer from the small-sized regulated firms to the large-sized regulated firms, which supports the reallocation effect.

#### 4.4.3. Target Market

The pollution haven hypothesis (PHH) suggests that capital and production of polluting industries shift to areas with less stringent environmental regulation, and domestic investment may be even more sensitive to spatial variation in environmental regulations than foreign investment [53], and exporters invest more in advanced abatement technologies [54]. With the exit of non-compliant firms, there are unoccupied orders and market share, and the existing firms choose between capturing the released market share and relocating to a more laxly regulated environment. In China, it usually takes two years for a factory to go from investment to production [22]. Given limited data, we use exporters in the pulp and paper industry to estimate the impact of SEL on firm income based on the types of the target market, including the local and overseas markets.

Before analyzing the impact of SEL on the heterogeneity of the regulated exporters, we compare the heterogeneous impact of this policy on the production scale and market performance between the local non-export firms and the exporters. Columns (1)–(4) of Table 8 are the DID results when the dependent variables are the logarithms of output and sales for the local firms and the exporters, respectively. When the dependent variables are the logarithm of output and the logarithm of sales, the interaction coefficients are statistically significant and negative for local firms, while those are insignificant for exporters. The results reveal that SEL in the Lake Tai area has a significant negative impact on local regulated firms, but has no significant impact on the total production scale and overall market performance of the exporters.

Columns (5)–(6) in Table 8 present the DID results of SEL on the local and overseas market sizes for the exporters. When the dependent variable is the exporters’ local market size, the coefficient of the interaction is insignificant and negative. The interaction coefficient is insignificant when the dependent variable is the exporters’ overseas market size. The results show that SEL’s implementation in the Lake Tai area has no significant impact on the overseas market scale and local market scale for the exporters.

**Figure 2:**
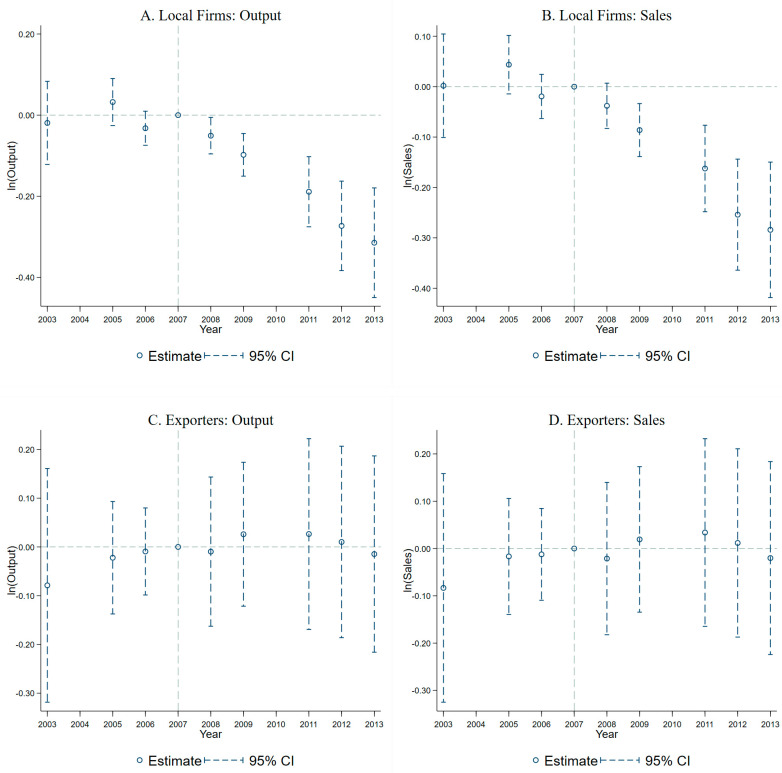
Local firms vs exporters: Production scale vs market scale.

**Figure 3:**
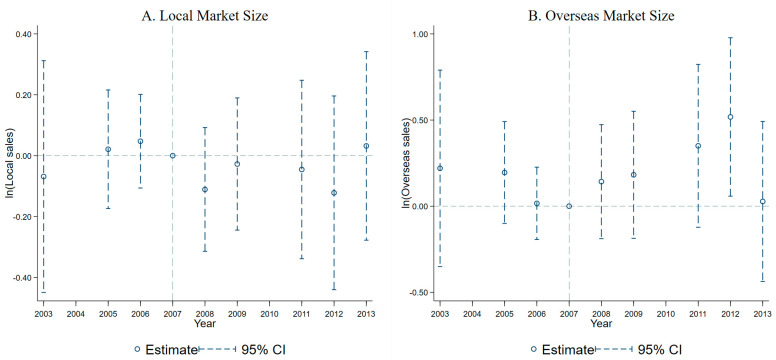
Exporters: Local market size vs overseas market size.

Figure 2 and Figure 3 show the results of the common trend test corresponding to the DID model of columns (1)–(4) and (5)–(6) in Table 8. The differences between the treatment and control groups for all six dependent variables are insignificant before 2008, indicating that the treatment and control groups followed a common trend from 2003 to 2007, and the causal effects in Table 8 are reliable.

The impact of SEL on the exporters’ production scale and market performance may be affected by firm size. Table 9 shows the results of the heterogeneity analysis for the exporters based on firm size. Among the export firms in the pulp and paper industry in the Lake Tai area, only the output and sales of large-sized exporters have significantly expanded, while the output and sales of medium and small-sized exporters have not been affected. We suppose that the production scale and market performance of the large-sized exporters rise rather than decline due to their stronger resource allocation capacity to absorb extra production from exited firms.

To test whether large-sized exporters have a stronger capacity to reallocate resources, we study the heterogeneity of different market bases affected by SEL based on firm size. Table 10 provides the results of the heterogeneity analysis based on the exporters’ size and market base. Compared with the control group, the coefficients of the 2008 SEL on the local market size and overseas market size are insignificant for the medium and small-sized exporters in the Lake Tai area. However, the 2008 SEL has a significant positive impact on the local market size for the large-sized exporters in the Lake Tai area, a significant negative impact on the overseas market size, and a significant positive impact on the overall market size. These findings imply that after SEL’s implementation in the Lake Tai area, the large-sized exporters pay more attention to the expansion of the local market size, while the medium and small-sized exporters pay more attention to maintaining the status quo. In other words, after facing more stringent environmental regulations, the reallocation effect of international markets is mainly reflected in the large-sized exporters.

## 5. Conclusions

This paper investigates the impact of the COD special emission limit on the production and market performance of firms in the pulp and paper industry in the Lake Tai area during 2003–2013. Using firm-level data and the difference-in-differences framework, we find that more stringent environmental regulations reduce firm performance. In particular, the production scale, profitability, and market size of firms in the pulp and paper industry in the Lake Tai area are less than those in the control group under the SEL. The implementation of SEL has heterogeneous effects on production scale and market performance across different types of regulated firms due to the reallocation effect of production. More production is transferred from non-SOE and small-sized to SOE and large-sized firms in the pulp and paper industry in more stringent counties than those in less stringent counties. We also find that the reallocation effect of global markets appears in exporting regulated firms, but mainly in large-sized firms due to the strong capacity in absorbing production re-leased by other regulated firms. In addition, the inventory alleviation effect plays an important role in environmental regulation impacting firm performance. Based on our empirical findings, this paper extends previous literature regarding the research scope and mechanisms.

Our findings have novel implications for policy-making. Although environmental regulations may degrade firm performance, the aim of environmental policies enacted by governments is a mix of environmental protection, technology upgrading, and sustainable economic growth, eventually improving welfare. The implementation of environmental regulation should set a buffer time for firms to comply and adapt. In addition, environmental policies should be enacted with different focuses on different types of firms. For instance, for medium-sized firms with limited liquidity, local governments should help introduce advanced technologies instead of shutting them down.

We acknowledge that this paper has several drawbacks. First, we do not have access to data about firms’ emissions to verify that SEL reduces firms’ COD emissions. This can be a future direction for the related topic to investigate. Second, due to the missing values of variables such as capital and immediate input, we can only estimate the effect of environmental regulation on firms’ direct performance instead of intrinsic productivity. However, with more comprehensive data, this line of research is expected to deepen the understanding of the production reallocation effect, such as interacting with the power of the market or government. In addition, several factors briefly discussed in this paper can be further investigated. Finally, future studies can focus on designing environmental regulation to achieve a trade-off between promoting technological upgrading and allowing more small-sized firms to survive.

## Figures and Tables

**Table 1:** Regulated regions in the Lake Tai area.

Province	City	District, County, and Prefecture-Level City	Implementation Time	Policy Source
Jiangsu	Suzhou	All municipal districts	1 September 2008	Ministry of Environmental Protection Announcement No. 28 of 2008
	Wuxi	All municipal districts		
	Changzhou	All municipal districts		
	Zhenjiang	Danyang City, Jurong City, and Dantu District		
	Nanjing	Lishui District and Gaochun County		
Zhejiang	Huzhou	All municipal districts		
	Jiaxing	All municipal districts		
	Hangzhou	Hangzhou city (Shangcheng District, Xiacheng District, Gongshu District, Jianggan District, Jianggan District, Yuhang District, and the area outside the Qiantang River basin in Xihu District) and the area outside Qiantang River basin in Lin’an City		
Shanghai		Qingpu District		
Shanghai		All districts except for Qingpu District	1 February 2009	Shanghai Municipal Government Announcement No. 52 of 2008

**Table 2:** Descriptive statistics.

Variable	Treatment Group				Control Group			
	2003–2007		2008–2013		2003–2007		2008–2013	
	Obs.	Mean	Obs.	Mean	Obs.	Mean	Obs.	Mean
ln (output)	1977	8.0229	2977	8.3325	6067	7.6649	7061	8.2649
ln (sales)	1977	8.0097	2976	8.3193	6068	7.6449	7060	8.2422
ln (income)	2629	7.9132	2979	8.3237	7647	7.5913	7063	8.2373
ln (export)	1977	1.3096	2656	1.5073	6069	0.6923	5939	0.9631

Note: The unit of output, sales, income, and export is 10,000 yuan.

**Table 3:** Baseline results.

	(1)	(2)	(3)	(4)
Dependent Variable	Ln (Output)	Ln (Sales)	Ln (Operating Income)	Ln (Exports)
Treat × Post	−0.095 ***	−0.091 ***	−0.080 ***	−0.099
	(0.026)	(0.026)	(0.028)	(0.092)
Size	0.360 ***	0.358 ***	0.387 ***	0.150 ***
	(0.019)	(0.019)	(0.019)	(0.037)
SOE	−0.017	−0.033	−0.024	0.097
	(0.093)	(0.093)	(0.087)	(0.260)
Age	0.000	0.000	0.000	0.004
	(0.002)	(0.002)	(0.002)	(0.007)
Constant	6.453 ***	6.444 ***	6.247 ***	0.323 *
	(0.088)	(0.088)	(0.088)	(0.183)
Year FE	Yes	Yes	Yes	Yes
Firm FE	Yes	Yes	Yes	Yes
County FE	Yes	Yes	Yes	Yes
R-squared	0.917	0.916	0.911	0.835
Observations	16,648	16,646	18,800	15,211

Note: Standard errors in parentheses are clustered at the firm level. *** and * denote significance at the 1% and 10% level, respectively.

**Table 4:** Robustness check: All 12 regulated industries as the treatment group.

	(1)	(2)	(3)	(4)
Dependent Variable	Ln (Output)	Ln (Sales)	Ln (Operating Income)	Ln (Exports)
Treat × Post	−0.249 ***	−0.246 ***	−0.263 ***	0.070
	(0.010)	(0.010)	(0.010)	(0.046)
ln (Labor)	0.331 ***	0.329 ***	0.359 ***	0.387 ***
	(0.006)	(0.006)	(0.006)	(0.018)
SOE	−0.053	−0.056	−0.044	0.012
	(0.040)	(0.042)	(0.040)	(0.155)
Age	0.002 **	0.002 **	0.001 *	0.013 ***
	(0.001)	(0.001)	(0.001)	(0.003)
Constant	6.544 ***	6.529 ***	6.330 ***	0.845 ***
	(0.030)	(0.030)	(0.030)	(0.093)
Year FE	Yes	Yes	Yes	Yes
Firm FE	Yes	Yes	Yes	Yes
County FE	Yes	Yes	Yes	Yes
R-squared	0.888	0.888	0.881	0.836
Observations	139,560	139,542	156,134	133,612

Notes: Standard errors in parentheses are clustered at the firm level. ***, ** and * denote significance at the 1%, 5%, and 10% level, respectively.

**Table 5:** Robustness check: Control for other fixed effects.

	(1)	(2)	(3)	(4)	(5)	(6)	(7)	(8)
Dependent Variable	Ln (Output)	Ln (Sales)	Ln (Operating Income)	Ln (Exports)	Ln (Output)	Ln (Sales)	Ln (Operating Income)	Ln (Exports)
Treat × Post	−0.169 ***	−0.169 ***	−0.170 ***	−0.054	−0.084 ***	−0.081 ***	−0.070 **	−0.095
	(0.031)	(0.031)	(0.033)	(0.087)	(0.026)	(0.026)	(0.028)	(0.092)
ln (Labor)	0.358 ***	0.356 ***	0.384 ***	0.152 ***	0.363 ***	0.360 ***	0.391 ***	0.145 ***
	(0.019)	(0.019)	(0.019)	(0.037)	(0.019)	(0.019)	(0.019)	(0.037)
SOE	−0.027	−0.043	−0.034	0.081	−0.028	−0.044	−0.032	0.085
	(0.089)	(0.088)	(0.081)	(0.258)	(0.092)	(0.092)	(0.085)	(0.260)
Age	0.001	0.001	0.001	0.004	−0.000	0.000	0.000	0.004
	(0.002)	(0.002)	(0.002)	(0.007)	(0.002)	(0.002)	(0.002)	(0.007)
Constant	6.468 ***	6.460 ***	6.269 ***	0.310 *	6.439 ***	6.432 ***	6.230 ***	0.344 *
	(0.087)	(0.088)	(0.087)	(0.184)	(0.088)	(0.088)	(0.088)	(0.184)
Province-year FE	Yes	Yes	Yes	Yes	No	No	No	No
CIC4-year FE	No	No	No	No	Yes	Yes	Yes	Yes
Firm FE	Yes	Yes	Yes	Yes	Yes	Yes	Yes	Yes
County FE	Yes	Yes	Yes	Yes	Yes	Yes	Yes	Yes
R-squared	0.919	0.918	0.914	0.836	0.918	0.917	0.912	0.836
Observations	16,648	16,646	18,800	15,211	16,646	16,644	18,798	15,209

Note: Standard errors in parentheses are clustered at the firm level. ***, ** and * denote significance at the 1%, 5%, and 10% level, respectively.

**Table 6:** Heterogeneity analysis: SOE vs non-SOE.

	(1)	(2)	(3)	(4)	(5)	(6)	(7)	(8)
Sample	SOE	SOE	SOE	SOE	Non-SOE	Non-SOE	Non-SOE	Non-SOE
Dependent Variable	Ln (Output)	Ln (Sales)	Ln (Operating Income)	Ln (Exports)	Ln (Output)	Ln (Sales)	Ln (Operating Income)	Ln (Exports)
Treat × Post	1.041 ***	1.010 ***	1.054 ***	1.307	−0.098 ***	−0.094 ***	−0.082 ***	−0.104
	(0.227)	(0.212)	(0.213)	(1.054)	(0.026)	(0.026)	(0.028)	(0.092)
Size	0.137	0.157	0.138	−1.282	0.361 ***	0.358 ***	0.387 ***	0.151 ***
	(0.149)	(0.153)	(0.135)	(1.162)	(0.019)	(0.019)	(0.019)	(0.037)
Age	0.006 *	0.006 *	0.002	0.002	0.000	0.000	0.000	0.004
	(0.003)	(0.003)	(0.002)	(0.004)	(0.002)	(0.002)	(0.002)	(0.008)
Constant	7.809 ***	7.702 ***	7.747 ***	7.444	6.449 ***	6.441 ***	6.243 ***	0.328 *
	(0.804)	(0.826)	(0.730)	(6.140)	(0.088)	(0.088)	(0.089)	(0.184)
Year FE	Yes	Yes	Yes	Yes	Yes	Yes	Yes	Yes
Firm FE	Yes	Yes	Yes	Yes	Yes	Yes	Yes	Yes
County FE	Yes	Yes	Yes	Yes	Yes	Yes	Yes	Yes
R-squared	0.982	0.983	0.974	0.852	0.917	0.916	0.911	0.836
Observations	92	92	127	88	16,522	16,520	18,637	15,089

Note: Standard errors in parentheses are clustered at the firm level. *** and * denote significance at the 1% and 10% level, respectively.

**Table 7:** Heterogeneity analysis: Firm scale.

	(1)	(2)	(3)	(4)	(5)	(6)	(7)	(8)	(9)
Sample	Large	Medium	Small	Large	Medium	Small	Large	Medium	Small
Dependent Variable	Ln (Output)	Ln (Output)	Ln (Output)	Ln (Sales)	Ln (Sales)	Ln (Sales)	Ln (Operating Income)	Ln (Operating Income)	Ln (Operating Income)
Treat × Post	0.385 ***	0.099	−0.118 ***	0.470 ***	0.072	−0.110 ***	0.562 ***	0.107	−0.106 ***
	(0.052)	(0.095)	(0.027)	(0.064)	(0.095)	(0.027)	(0.092)	(0.106)	(0.029)
Size	0.730 ***	0.294 ***	0.360 ***	0.792 ***	0.294 ***	0.359 ***	0.423 **	0.382 ***	0.386 ***
	(0.116)	(0.081)	(0.021)	(0.114)	(0.082)	(0.021)	(0.175)	(0.086)	(0.021)
Age	0.005 **	0.010	−0.000	0.007 ***	0.010	−0.000	0.001	0.009	−0.000
	(0.002)	(0.008)	(0.002)	(0.002)	(0.008)	(0.002)	(0.004)	(0.007)	(0.002)
Constant	6.432 ***	8.154 ***	6.297 ***	5.845 ***	8.147 ***	6.283 ***	8.782 ***	7.563 ***	6.103 ***
	(0.963)	(0.513)	(0.093)	(0.955)	(0.516)	(0.094)	(1.419)	(0.538)	(0.094)
Year FE	Yes	Yes	Yes	Yes	Yes	Yes	Yes	Yes	Yes
Firm FE	Yes	Yes	Yes	Yes	Yes	Yes	Yes	Yes	Yes
County FE	Yes	Yes	Yes	Yes	Yes	Yes	Yes	Yes	Yes
R-squared	0.969	0.899	0.882	0.969	0.899	0.880	0.969	0.880	0.874
Observations	60	1210	14,339	60	1210	14,337	66	1339	16,383

Note: Standard errors in parentheses are clustered at the firm level. *** and ** denote significance at the 1% and 5% level, respectively.

**Table 8:** Heterogeneity analysis: Local firms vs exporters.

	(1)	(2)	(3)	(4)	(5)	(6)
Sample	Local Firms	Local Firms	Exporters	Exporters	Exporters	Exporters
Dependent Variable	Ln (Output)	Ln (Sales)	Ln (Output)	Ln (Sales)	Local Market Size	Overseas Market Size
Treat × Post	−0.115 ***	−0.108 ***	0.025	0.021	−0.066	0.150
	(0.027)	(0.027)	(0.074)	(0.074)	(0.098)	(0.163)
Size	0.374 ***	0.373 ***	0.285 ***	0.280 ***	0.488 ***	0.373 ***
	(0.021)	(0.021)	(0.047)	(0.048)	(0.111)	(0.092)
SOE	−0.056	−0.076	0.102	0.062	0.012	−0.165
	(0.109)	(0.108)	(0.246)	(0.231)	(0.190)	(0.234)
Age	−0.001	−0.001	0.003	0.003	0.003	0.005
	(0.002)	(0.002)	(0.003)	(0.003)	(0.003)	(0.008)
Constant	6.273 ***	6.259 ***	7.293 ***	7.303 ***	5.825 ***	5.034 ***
	(0.096)	(0.095)	(0.258)	(0.262)	(0.599)	(0.493)
Year FE	Yes	Yes	Yes	Yes	Yes	Yes
Firm FE	Yes	Yes	Yes	Yes	Yes	Yes
County FE	Yes	Yes	Yes	Yes	Yes	Yes
R-squared	0.909	0.908	0.948	0.946	0.928	0.817
Observations	12,828	12,827	3258	3256	1894	2076

Note: Standard errors in parentheses are clustered at the firm level. *** denotes significance at the 1% level.

**Table 9:** Heterogeneity analysis: Firm Size of Exporters in Production Size and Market Size.

	(1)	(2)	(3)	(4)	(5)	(6)
Sample	Exporters & Large	Exporters & Medium	Exporters & Small	Exporters & Large	Exporters & Medium	Exporters & Small
Dependent Variable	Ln (Output)	Ln (Output)	Ln (Output)	Ln (Sales)	Ln (Sales)	Ln (Sales)
Treat × Post	0.324 ***	0.115	0.007	0.414 ***	0.096	0.007
	(0.081)	(0.152)	(0.092)	(0.099)	(0.151)	(0.093)
Size	0.701 ***	0.244	0.276 ***	0.811 ***	0.248 *	0.271 ***
	(0.185)	(0.149)	(0.056)	(0.203)	(0.148)	(0.057)
Age	0.006 ***	−0.002	0.006	0.009 ***	−0.004	0.005
	(0.001)	(0.055)	(0.004)	(0.002)	(0.054)	(0.004)
Constant	6.683 ***	8.770 ***	7.021 ***	5.697 ***	8.745 ***	7.034 ***
	(1.539)	(1.147)	(0.289)	(1.689)	(1.136)	(0.294)
Year FE	Yes	Yes	Yes	Yes	Yes	Yes
Firm FE	Yes	Yes	Yes	Yes	Yes	Yes
County FE	Yes	Yes	Yes	Yes	Yes	Yes
R-squared	0.959	0.943	0.904	0.959	0.945	0.901
Observations	50	481	2356	50	481	2354

Note: Standard errors in parentheses are clustered at the firm level. *** and * denote significance at the 1% and 10% level, respectively.

**Table 10:** Heterogeneity analysis: Firm size of exporters in local market size and overseas market size.

	(1)	(2)	(3)	(4)	(5)	(6)
Sample	Exporters & Large	Exporters & Medium	Exporters & Small	Exporters & Large	Exporters & Medium	Exporters & Small
Dependent Variable	Local Market Size	Local Market Size	Local Market Size	Overseas Market Size	Overseas Market Size	Overseas Market Size
Treat × Post	0.397 ***	0.033	−0.064	−1.443 *	−0.006	0.244
	(0.123)	(0.183)	(0.125)	(0.663)	(0.251)	(0.202)
Size	0.801 **	0.593 *	0.459 ***	1.025	0.472 **	0.359 ***
	(0.306)	(0.313)	(0.128)	(1.701)	(0.230)	(0.107)
Age	0.012 ***	0.010	0.007	0.002	−0.064	0.011
	(0.002)	(0.054)	(0.006)	(0.013)	(0.105)	(0.013)
Constant	5.550*	5.971 ***	5.580 ***	1.916	5.815 ***	4.825 ***
	(2.536)	(2.131)	(0.642)	(14.130)	(2.059)	(0.523)
Year FE	Yes	Yes	Yes	Yes	Yes	Yes
Firm FE	Yes	Yes	Yes	Yes	Yes	Yes
County FE	Yes	Yes	Yes	Yes	Yes	Yes
R-squared	0.932	0.926	0.883	0.752	0.849	0.780
Observations	49	390	1365	49	398	1531

Note: Standard errors in parentheses are clustered at the firm level. ***, ** and * denote significance at the 1%, 5%, and 10% level, respectively.

## Data Availability

The data presented in this study are available on request from the corresponding author. The data are not publicly available due to privacy.

## Appendix A

**Table A1:** COD emission limits for the pulp and paper industry in Jiangsu Province (Unit: mg/L).

Production Process			From 1 January 2001	From 1 January 2004 (Firms Established Since 1 January 2004)	From 1 January 2005 (Firms Established before 1 January 2004)	From 1 January 2008 (Local Standard)	From 1 January 2008 (Firms Established since 1 August 2008)	1 May 2009–30 January 2011 (Firms Established before 1 August 2008)	From 1 July 2011 (Firms Established before 1 August 2008	From 1 September 2008 (SEL’s Implementation)
Pulp	Wood pulp pulping	Natural color	350				100	200	100	80
		Bleaching	400							
	Straw pulp pulping	Natural color	400							
		Bleaching	450							
	Waste paper pulping	Natural color	400	-	-					
		Deinking	450	-	-					
Pulp and paper	Wood pulp pulping	Natural color	350				90	150	90	60
		Bleaching	400							
	Straw pulp pulping	Natural color	400							
		Bleaching	450							
	Waste paper pulping	Natural color	400	100	100	100		120		
		Deingking	450	150	150					
Paper making	Waste paper		100			100	80	100	80	50
	Commercial pulp					80				

Note: This table reports the national standard for discharge except for the noted.

**Table A2:** Classification criteria for firm size.

Firm Size	2003 Standard			2011 Standard		
	Number of Employees	Sales (Million Yuan)	Total Asset (Million Yuan)		Number of Employees	Revenue from Main Business (Million Yuan)
Large	[2000, +∞)	[30,000, +∞)	[40,000, +∞)		[1000, +∞)	[40,000, +∞)
Medium	[300, 2000)	[3000, 30,000)	[4000, 40,000)		[300, 1000)	[2000, 40,000)
Small	(0, 300)	(0, 3000)	(0, 4000)		[20, 300)	[300, 2000)
Micro					(0, 20)	(0, 300)

Note: This table is organized according to the National Statistics [2003] No.17 and the National Statistics [2011] No.75.

## References

[1] Fang Z., Huang B., Yang Z. (2020). Trade openness and the environmental Kuznets curve: Evidence from Chinese cities. World Econ..

[2] Wang M., Webber M., Finlayson B., Barnett J. (2008). Rural industries and water pollution in China. J. Environ. Manag..

[3] Zheng S., Kahn M.E. (2017). A New Era of Pollution Progress in Urban China?. J. Econ. Perspect..

[4] Cai H., Chen Y., Gong Q. (2016). Polluting thy neighbor: Unintended consequences of China׳s pollution reduction mandates. J. Environ. Econ. Manag..

[5] Li Y., Lin F., Wang W. (2022). Environmental regulation and inward foreign direct investment: Evidence from the eleventh Five-Year Plan in China. J. Econ. Surv..

[6] Wu H., Guo H., Zhang B., Bu M. (2017). Westward movement of new polluting firms in China: Pollution reduction mandates and location choice. J. Comp. Econ..

[7] Zhou L., Li L.-Z., Huang J.-K. (2021). The river chief system and agricultural non-point source water pollution control in China. J. Integr. Agric..

[8] Li J., Shi X., Wu H., Liu L. (2020). Trade-off between economic development and environmental governance in China: An analysis based on the effect of river chief system. China Econ. Rev..

[9] She Y., Liu Y., Jiang L., Yuan H. (2019). Is China’s River Chief Policy effective? Evidence from a quasi-natural experiment in the Yangtze River Economic Belt, China. J. Clean. Prod..

[10] He G., Wang S., Zhang B. (2020). Watering Down Environmental Regulation in China. Q. J. Econ..

[11] Kahn M.E., Li P., Zhao D. (2015). Water Pollution Progress at Borders: The Role of Changes in China’s Political Promotion Incentives. Am. Econ. J. Econ. Policy.

[12] Wang H., Fan C., Chen S. (2021). The impact of campaign-style enforcement on corporate environmental Action: Evidence from China’s central environmental protection inspection. J. Clean. Prod..

[13] Zhang B., Chen X., Guo H. (2018). Does central supervision enhance local environmental enforcement? Quasi-experimental evidence from China. J. Public Econ..

[14] Liu M., Shadbegian R., Zhang B. (2017). Does environmental regulation affect labor demand in China? Evidence from the textile printing and dyeing industry. J. Environ. Econ. Manag..

[15] Zhang Y., Cui J., Lu C. (2020). Does environmental regulation affect firm exports? Evidence from wastewater discharge standard in China. China Econ. Rev..

[16] Chakraborti L. (2016). Do plants’ emissions respond to ambient environmental quality? Evidence from the clean water act. J. Environ. Econ. Manag..

[17] Wang C., Wu J., Zhang B. (2018). Environmental regulation, emissions and productivity: Evidence from Chinese COD-emitting manufacturers. J. Environ. Econ. Manag..

[18] Zhang C., Tao R., Yue Z., Su F. (2023). Regional competition, rural pollution haven and environmental injustice in China. Ecol. Econ..

[19] Shapiro J.S., Walker R. (2018). Why Is Pollution from US Manufacturing Declining? The Roles of Environmental Regulation, Productivity, and Trade. Am. Econ. Rev..

[20] Chen Y., Cheng L., Lee C.-C. (2022). How does the use of industrial robots affect the ecological footprint? International evidence. Ecol. Econ..

[21] Yu J., Shi X., Guo D., Yang L. (2021). Economic policy uncertainty (EPU) and firm carbon emissions: Evidence using a China provincial EPU index. Energy Econ..

[22] Chen Z., Kahn M.E., Liu Y., Wang Z. (2018). The consequences of spatially differentiated water pollution regulation in China. J. Environ. Econ. Manag..

[23] Walker W.R. (2011). Environmental Regulation and Labor Reallocation: Evidence from the Clean Air Act. Am. Econ. Rev..

[24] Zheng J., He J., Shao X., Liu W. (2022). The employment effects of environmental regulation: Evidence from eleventh five-year plan in China. J. Environ. Manag..

[25] Bo S. (2021). Environmental Regulations, Political Incentives and Local Economic Activities: Evidence from China. Oxf. Bull. Econ. Stat..

[26] Wang Q., Zhu L. (2021). Environmental regulation, firm heterogeneity, and intra-industry reallocation. China Econ. Rev..

[27] Chen Q., Chen Z., Liu Z., Serrato J.C., Xu D. (2021). Regulating Conglomerates in China: Evidence from an Energy Conservation Program. National Bureau of Economic Research. https://www.nber.org/papers/w29066.

[28] Wang H., Wang C., Wu W., Mo Z., Wang Z. (2003). Persistent organic pollutants in water and surface sediments of Taihu Lake, China and risk assessment. Chemosphere.

[29] Zhao G., Gao J., Tian P., Tian K., Ni G. (2011). Spatial–temporal characteristics of surface water quality in the Taihu Basin, China. Environ. Earth Sci..

[30] Ebenstein A. (2012). The Consequences of Industrialization: Evidence from Water Pollution and Digestive Cancers in China. Rev. Econ. Stat..

[31] Wang Q., Yang Z. (2016). Industrial water pollution, water environment treatment, and health risks in China. Environ. Pollut..

[32] Zhou Z., Liu J., Zhou N., Zhang T., Zeng H. (2021). Does the “10-Point Water Plan” reduce the intensity of industrial water pollution? Quasi-experimental evidence from China. J. Environ. Manag..

[33] Zohdi E., Abbaspour M. (2019). Harmful algal blooms (red tide): A review of causes, impacts and approaches to monitoring and prediction. Int. J. Environ. Sci. Technol..

[34] Blackman A., Li Z., Liu A.A. (2018). Efficacy of Command-and-Control and Market-Based Environmental Regulation in Developing Countries. Annu. Rev. Resour. Econ..

[35] Nian H., Wang C., Yin H. (2022). Size control or intensity control: A comparative study of two Common Environmental Regulations. J. Regul. Econ..

[36] Brandt L., Van Biesebroeck J., Zhang Y. (2012). Creative accounting or creative destruction? Firm-level productivity growth in Chinese manufacturing. J. Dev. Econ..

[37] Chen Y., Jiang H., Liang Y., Pan S. (2022). The impact of foreign direct investment on innovation: Evidence from patent filings and citations in China. J. Comp. Econ..

[38] Jiang H., Pan S., Ren X. (2020). Does Administrative Approval Impede Low-Quality Innovation? Evidence from Chinese Manufacturing Firms. Sustainability.

[39] Pan X., Pu C., Yuan S., Xu H. (2022). Effect of Chinese pilots carbon emission trading scheme on enterprises’ total factor productivity: The moderating role of government participation and carbon trading market efficiency. J. Environ. Manag..

[40] Lim K.Y., Morris D. (2022). Thresholds in natural resource rents and state owned enterprise profitability: Cross country evidence. Energy Econ..

[41] Coad A., Holm J.R., Krafft J., Quatraro F. (2018). Firm age and performance. J. Evol. Econ..

[42] Jiang L., Zhou H., He S. (2021). Does energy efficiency increase at the expense of output performance: Evidence from manufacturing firms in Jiangsu province, China. Energy.

[43] Thornhill S. (2006). Knowledge, innovation and firm performance in high- and low-technology regimes. J. Bus. Ventur..

[44] Sun S., Jiang H. (2022). CEO turnover and corporate innovation: What can we learn from Chinese listed companies. Front. Psychol..

[45] Tombe T., Winter J. (2015). Environmental policy and misallocation: The productivity effect of intensity standards. J. Environ. Econ. Manag..

[46] Anand K., Anupindi R., Bassok Y. (2008). Strategic Inventories in Vertical Contracts. Manag. Sci..

[47] Dey K., Roy S., Saha S. (2019). The impact of strategic inventory and procurement strategies on green product design in a two-period supply chain. Int. J. Prod. Res..

[48] Andersson F.N.G., Opper S., Khalid U. (2018). Are capitalists green? Firm ownership and provincial CO emissions in China. Energy Policy.

[49] Cai X., Zhu B., Zhang H., Li L., Xie M. (2020). Can direct environmental regulation promote green technology innovation in heavily polluting industries? Evidence from Chinese listed companies. Sci. Total Environ..

[50] Li M., Sun X., Wang Y., Song-Turner H. (2019). The impact of political connections on the efficiency of China’s renewable energy firms. Energy Econ..

[51] Fan H., Peng Y., Wang H., Xu Z. (2021). Greening through finance?. J. Dev. Econ..

[52] Qi J., Tang X., Xi X. (2021). The Size Distribution of Firms and Industrial Water Pollution: A Quantitative Analysis of China. Am. Econ. J. Macroecon..

[53] Millimet D.L., Roy J. (2016). Empirical Tests of the Pollution Haven Hypothesis When Environmental Regulation is Endogenous. J. Appl. Econ..

[54] Kwon O., Zhao H., Zhao M.Q. (2023). Global firms and emissions: Investigating the dual channels of emissions abatement. J. Environ. Econ. Manag..

